# RETRACTION: Shame in Patients Undergoing Ureterostomy: A Cross‐Sectional Survey

**DOI:** 10.1111/iwj.70662

**Published:** 2025-04-23

## Abstract

**RETRACTION:** LiQ. , ZhuoL. , and ZhangT. , “Shame in Patients Undergoing Ureterostomy: A Cross‐Sectional Survey,” International Wound Journal 21, no. 3 (2024): e14793, 10.1111/iwj.14793.38453161 PMC10920030

The above article, published online on 07 March 2024, in Wiley Online Library (http://onlinelibrary.wiley.com/), has been retracted by agreement between the journal Editor in Chief, Professor Keith Harding; and John Wiley & Sons Ltd. Following an investigation by the publisher, all parties have concluded that this article was accepted solely on the basis of a compromised peer review process. The editors have therefore decided to retract the article. The authors did not respond to our notice regarding the retraction.

**RETRACTION:** Q. Li , L. Zhuo , and T. Zhang , “Shame in Patients Undergoing Ureterostomy: A Cross‐Sectional Survey,” International Wound Journal 21, no. 3 (2024): e14793, 10.1111/iwj.14793.38453161 PMC10920030

The above article, published online on 07 March 2024, in Wiley Online Library (http://onlinelibrary.wiley.com/), has been retracted by agreement between the journal Editor in Chief, Professor Keith Harding; and John Wiley & Sons Ltd. Following an investigation by the publisher, all parties have concluded that this article was accepted solely on the basis of a compromised peer review process. The editors have therefore decided to retract the article. The authors did not respond to our notice regarding the retraction.

